# Welcome to *PathoGenetics*

**DOI:** 10.1186/1755-8417-1-1

**Published:** 2008-11-03

**Authors:** Andrea Ballabio, Stylianos Antonarakis

**Affiliations:** 1 Telethon Institute of Genetics and Medicine, Napoli, Italy; 2 Department of Genetic Medicine and Development, University of Geneva Medical School, and University Hospitals of Geneva, Geneva, Switzerland

## Abstract

Disease gene identification has made enormous strides in the past twenty years through functional, positional and candidate gene approaches, and more recently by the exploitation of genome-wide strategies. However, although pathogenic mutations in over 2000 genes have been identified as causative of human diseases, much less is known about the relationship between the molecular defects and mechanisms that lead to disease pathology and symptoms. Recent advances in diverse fields such as genomics, proteomics, cell biology, as well as studies on transgenic animals have greatly accelerated our understanding of the biochemical and cellular basis of many diseases but much still remains to be discovered. The current challenge is to understand the molecular and metabolic pathways by which a particular pathogenic variation leads to a specific phenotype. The study of abnormal conditions is of crucial importance for the understanding of normal physiology and often provides us with the rationale for the development of novel therapeutic strategies.

## 

*PathoGenetics *was created to meet the needs of the scientific community for a journal focused solely on the **patho**genesis of **genetic **diseases. Currently studies on disease pathogenesis can be found in a variety of different journals with different scopes but none focuses entirely on the molecular and metabolic pathways perturbed in genetic diseases. *PathoGenetics *is a multidisciplinary journal which welcomes submissions from different approaches including cell biology, biochemistry, developmental and systems biology. Given its unique characteristics, *PathoGenetics *is likely to become the ideal journal for scientists from different backgrounds to publish and read exciting research on disease pathogenesis. *PathoGenetics *focuses on both *in vitro *and *in vivo *studies on the cascade of events leading from genomic lesions (i.e. either a gene mutation or a genomic rearrangement) to disease phenotypes. The discovery of novel molecular and metabolic pathways relevant to disease pathogenesis will be given specific emphasis.

The open access publishing model is the most effective way of ensuring that the research we publish can be accessed, read and built upon [1]. Once an article is accepted it is immediately published and deposited in PubMed ensuring dissemination of every article to a large audience independent of an access to a journal subscription and therefore directly benefiting the scientific community. There is evidence that an open access article is more likely to be used and cited than one subject to subscription barriers [2]. Manuscripts submitted to *PathoGenetics *will be peer-reviewed by at least two experts in the field after initial screening by the Editors-in-Chief and a consultation from the editorial board. We are delighted and proud that *Pathogenetics *has an outstanding editorial board [3], who will work with us to ensure the high quality of the research published in this new journal.

We are committed to making this journal a success and believe *PathoGenetics *will give scientists the unique opportunity to publish exciting research on the molecular mechanisms underlying the manifestation of disease phenotypes and provide a forum for the understanding of the biochemical and cellular mechanisms that underlie genetic disease. We would like to take this opportunity to welcoming you to this exciting new journal, and invite you to submit your important work to *PathoGenetics*.

## Competing interests

The authors declare that they have no competing interests.

## References

[B1] BioMed Central Open Access Charter. http://www.biomedcentral.com/info/about/charter.

[B2] Eysenbach G (2006). Citation advantage of open access articles. PloS Biol.

[B3] PathoGenetics Editorial Board. http://www.pathogeneticsjournal.com/edboard/.

